# Influenza Vaccination Practices of Physicians and Caregivers of Children with Neurologic and Neurodevelopmental Conditions — United States, 2011–12 Influenza Season

**Published:** 2013-09-13

**Authors:** Michael J. Smith, Deborah McFalls, Jennifer Hendricks, Janice Watkins, Cynthia Moore, Georgina Peacock, Adina de Coteau

**Affiliations:** Univ of Louisville School of Medicine, Louisville, Kentucky; Oak Ridge Associated Universities, Oak Ridge, Tennessee; Div of Birth Defects and Developmental Disabilities, National Center on Birth Defects and Developmental Disabilities, CDC

Cognitive dysfunction, seizure disorders (epilepsy), and other neurologic disorders are conditions associated with a high risk for complications of influenza virus infection (1–3). This risk was observed during the 2009 influenza pandemic; among 336 pediatric deaths, 146 occurred in children with underlying neurologic disorders, most commonly intellectual disability (76%) and epilepsy (51%) (4). Because little is known about influenza-related knowledge and practices among the families and health-care providers of children with neurologic or neurodevelopmental (NND) conditions, CDC worked with Family Voices and the American Academy of Pediatrics to survey parents and physicians during the 2011–12 influenza season to assess these factors. Among 1,005 children with NND conditions, parents reported that 50% of children were vaccinated or had a vaccine appointment scheduled. Vaccination rates were low for children with intellectual disability (52%) and epilepsy (59%). Physician recognition of high-risk conditions was low for intellectual disability (46%) and epilepsy (52%). Efforts to improve physician awareness are essential because physicians are in a key position to educate parents of children with NND conditions about their increased risk for influenza complications and the importance of prevention through vaccination. Further research also is needed to identify barriers to influenza vaccination among families and health-care providers of these children.

CDC collaborated with Family Voices, a national advocacy group for children with special health-care needs, to recruit via listservs parents of children with chronic medical conditions. An online survey was distributed to members of the Family Voices listservs and administered from September 6 through October 24, 2011. Parents or other caregivers were asked about their knowledge, attitudes, and practices related to having their children vaccinated with seasonal influenza vaccine.

This report focuses on vaccination behavior during the 2011–12 influenza season. For purposes of this study, vaccination rates were calculated by dividing the number of children reported to have been vaccinated or for whom a vaccination appointment was scheduled by the number of children for whom a response was obtained. Only children aged ≥6 months with high-risk conditions as defined by the Advisory Committee on Immunization Practices (1) were included in the analysis.

CDC also collaborated with the American Academy of Pediatrics to recruit primary-care and specialty physicians who provide care for children at high risk for influenza complications, specifically children with neurologic conditions. Physicians were recruited through American Academy of Pediatrics specialty listservs, including the Council on Children with Disabilities, the Committee on Practice and Ambulatory Medicine, and the Section on Neurology. An online survey was available from March 7 through May 15, 2011. This survey collected basic information regarding practice setting, specialty, and vaccination practices for various patient populations. Respondents also were asked which chronic medical conditions were associated with increased risk for severe illness from influenza.

Descriptive statistics were summarized as percentages. Between-group differences were assessed using chi-square testing. A p-value of <0.05 was considered to be statistically significant.

A total of 1,940 surveys were completed by parents of children with a high-risk condition. Seasonal influenza vaccination rates categorized by high-risk condition ranged from 41% for children with metabolic conditions to 78% for children with chronic lung disease (Figure). Among 1,005 parents of children with NND conditions, 50% reported their child had received or had an appointment scheduled to receive influenza vaccine at the time of survey completion.

Among all respondents, health-care providers were reported most frequently (75%) as the source of information about vaccines in general, and influenza vaccine specifically. Parents of vaccinated children were more likely (80% versus 64% [p<0.001]) to report using health-care providers as a source of information. Use of the Internet (24%) and family support or disability advocacy organizations (22%) were less frequently reported as sources of information and did not differ between families of vaccinated and unvaccinated children.

A total of 412 physicians participated in the online survey. Of those, 183 (44%) respondents identified themselves as primary-care providers. Among the remaining physicians, the predominant specialties were neurology (65), emergency medicine (56), critical care (28) and genetics/metabolism (24). A total of 393 physicians completed the question about high-risk conditions (Figure). Intellectual disability and hematologic disorders other than sickle cell disease were considered to be high-risk conditions by a minority of respondents. Further analyses were performed on a subset of physicians most likely to provide outpatient medical care to children with NND conditions: primary-care pediatricians, neurologists, geneticists, developmental pediatricians, and physiatrists. These physicians caring for children with NND conditions were more likely than other pediatricians to indicate cerebral palsy (79% versus 63%), epilepsy (57% versus 39%), spinal cord conditions (76% versus 60%), stroke (63% versus 41%), and other brain conditions (62% versus 44%) as high-risk conditions (p<0.05 for all comparisons). They were not more likely to rate intellectual disability as a high-risk condition.

What is already known on this topic?

Since 2005, the Advisory Committee on Immunization Practices has included cognitive dysfunction, spinal cord injuries, seizure disorders, and other neuromuscular disorders as high-risk conditions for complications associated with influenza virus infection. A review of pediatric influenza deaths during the 2009 H1N1 pandemic revealed that 146 (43%) of the 336 deaths occurred in children with an underlying neurologic condition.

What is added by this report?

Parents of children with neurologic or neurodevelopmental disorders and physicians caring for such children were surveyed by CDC during the 2011–12 influenza season. Parents responding to an online survey reported that 50% of 1,005 children with a neurologic disorder were vaccinated against influenza or had a vaccine appointment scheduled. Among the physicians, intellectual disability was recognized as a high-risk condition by 46% of respondents and epilepsy by 52%.

What are the implications for public health practice?

Vaccination coverage levels among children with neurologic conditions are comparable with those of healthy children, despite the fact that they are at increased risk for poor outcomes. Further research among families and health-care providers is needed to identify barriers to influenza immunization.

## Editorial Note

Annual influenza vaccination is recommended for all children aged 6 months–18 years. Although they are at greater risk for poor outcomes related to infection with influenza viruses, influenza vaccination of children with NND conditions was similar to that observed in the general pediatric population. The results of this survey are consistent with 2011–12 national seasonal influenza vaccination coverage estimates of 52% among children aged 6 months–17 years in the general population (5). In contrast, the *Healthy People 2020* goal (IID-12) is to increase the percentage of children who are vaccinated annually to 80% (6). Parents and caregivers reported that health-care providers were the most important source of information about vaccines. Intellectual disability and epilepsy were the two most common NND conditions among children who died during the 2009 influenza pandemic (2) but were two of the three conditions least likely to be recognized as high-risk by physicians.

The findings in this report are subject to at least four limitations. First, selection bias likely affected the results. Both the health-care provider and caregiver surveys were distributed via listserv services that require a subscription, which might have led to the exclusion of non–American Academy of Pediatrics member physicians who treat children at high risk for influenza complications. In addition, physicians especially interested in influenza prevention and treatment might be overrepresented in the sample. Similarly, the caregiver survey excludes parents and caregivers who are not on Family Voices listservs. Second, although it was not possible to calculate response rates because both surveys were distributed to multiple listservs, participation bias also likely affected the results. Third, the results of both surveys are based on self-report and might not reflect actual vaccination practices. Also, because this study assessed parental intent to vaccinate and parents were surveyed early in the influenza season, current vaccination and scheduled vaccination appointments were combined. However, parental intent to vaccinate a child might not have always resulted in vaccination. Finally, the physicians who participated in the health-care provider survey were not the same physicians who treated the patients in the parent survey. Therefore, their responses might not be representative of the experiences the caregivers had with their own health-care providers.

Despite these limitations, the results of these surveys demonstrate that children with NND conditions are no more likely to be vaccinated than healthy children, despite the fact that they are at increased risk for poor outcomes. Health-care providers remain the primary source of information regarding influenza vaccination. Increased outreach and communication efforts to both primary- and subspecialty-care providers might help reduce influenza-related morbidity and mortality among these children.

## Figures and Tables

**FIGURE:**
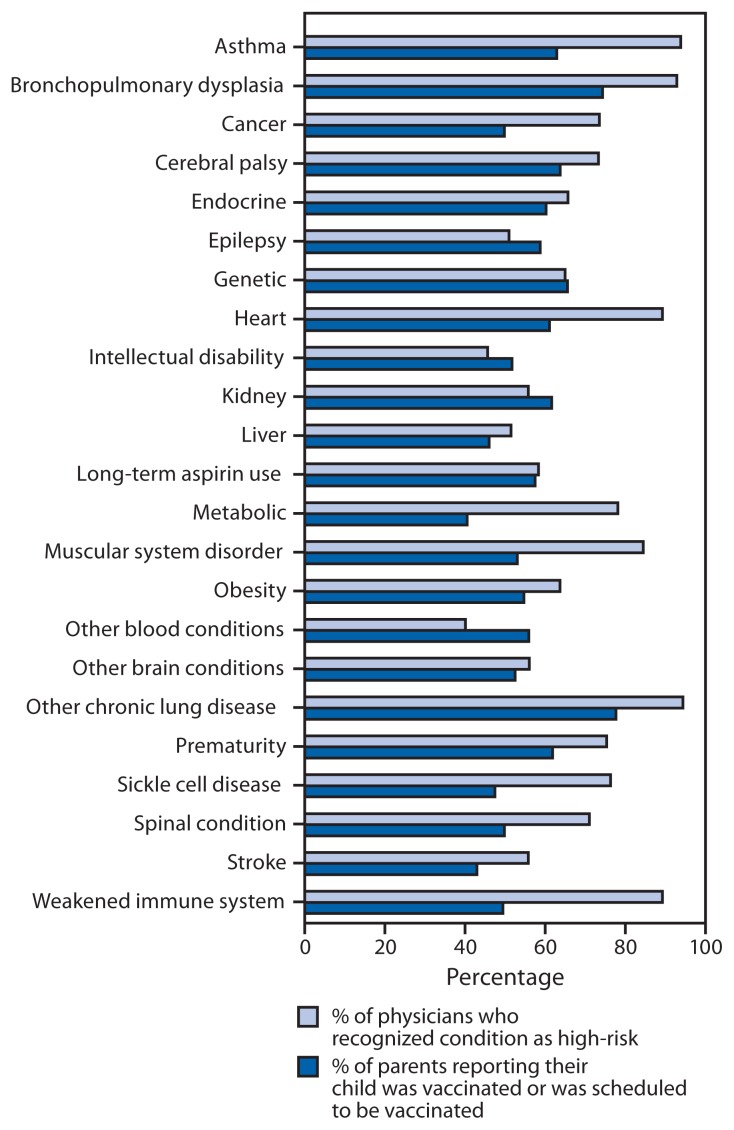
Influenza vaccination coverage among children at high risk for complications of influenza and physician recognition of high-risk conditions — United States, 2010–11 influenza season
